# Establishing the performance and acceptability of dried blood spot sampling to screen for islet‐specific autoantibodies

**DOI:** 10.1111/dme.70071

**Published:** 2025-05-19

**Authors:** Siân E. Faustini, Lauren M. Quinn, Madeeha Hoque, Siobhan Young, Christopher Bentley, Hin‐Fai Kwok, Timothy Plant, Ian Litchfield, Felicity Boardman, Sheila M. Greenfield, Parth Narendran, Alex G. Richter

**Affiliations:** ^1^ Clinical Immunology Services University of Birmingham Birmingham UK; ^2^ College of Medicine and Health University of Birmingham Birmingham UK; ^3^ Department of Immunology and Immunotherapy University of Birmingham Birmingham UK; ^4^ Institute of Applied Health Research University of Birmingham Birmingham UK; ^5^ Applied Health Directorate, Warwick Medical School University of Warwick Coventry UK; ^6^ Department of Diabetes University Hospitals of Birmingham Birmingham UK

**Keywords:** autoantibodies, disease prediction, GADA, IA‐2A, screening, type 1 diabetes, ZnT8A

## Abstract

**Background:**

Islet‐specific autoantibodies predate and predict the onset of type 1 diabetes and can be used to screen for presymptomatic disease. Dried blood spots (DBS) offer a convenient and reliable method for community‐based capillary sampling requiring low blood volumes compared to venous collection. We aimed to verify the use of DBS for detecting autoantibodies by the ElisaRSR (3‐screen) multiplex assay compared to venous sampling and also explore the acceptability of DBS sampling.

**Methods:**

Paired serum and DBS samples were collected from healthy controls (HC) and individuals with type 1 diabetes on insulin. Validation and verification of a 3‐screen Islet cell autoantibody (IA‐2A, GADA and ZnT8A) ELISA assay was undertaken for both matrices and compared. Perceived acceptability of DBS testing was explored via semi‐structured interviews with parents and professional stakeholders.

**Results:**

Temporally paired serum and DBS samples were collected for 101 individuals with type 1 diabetes (aged 7–73 years) and 22 HC (aged 18–60 years). Performance characteristics were similar for serum and DBS; sensitivity for serum was 86% compared to 89% for DBS and a specificity of 97% for serum compared to 100% for DBS. Parents (*n* = 38) and stakeholders (*n* = 25) thought DBS testing offered a minimally invasive, convenient screening test. Parents emphasised choice of screening location, including home and community settings.

**Conclusions:**

DBS sampling can be used as an alternative to serum for use with the 3‐screen assay for general population type 1 diabetes autoantibody screening. DBS sampling appears acceptable to parents and stakeholders.

## Graphical abstract

This comprehensive validation and acceptability study shows dried blood spot sampling can be used as an alternative to serum for use with the 3‐screen assay for population type 1 diabetes autoantibody screening and appears acceptable to parents and stakeholders.

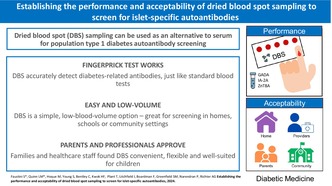

**What is already known?**

- Islet‐specific autoantibodies predate and predict the onset of type 1 diabetes and can be used to screen for presymptomatic disease.

**What this study has found?**

- This comprehensive validation and acceptability study shows dried blood spot (DBS) sampling can be used as an alternative to serum for use with the 3‐screen assay for population type 1 diabetes autoantibody screening and appears acceptable to parents and stakeholders.

**What are the implications of the study?**

- DBS sampling with the 3‐screen assay offers multiple advantages as a screening test, including lower blood volume requirement, low failure rate, high accessibility, lower cost, and is amenable to high‐throughput testing for population screening.

## 1 INTRODUCTION

Islet‐specific autoantibodies (Aab) predict and predate clinical onset of type 1 diabetes (T1D).^1^ The four main routinely measured islet cell Aab are anti‐insulin (IAA), anti‐glutamic acid decarboxylase (GADA), anti‐insulinoma‐associated autoantigen 2 (IA‐2A), and anti‐zinc transporter 8 (ZnT8A).^2^ Aab are present in approximately 85% of cases at symptomatic onset and predate clinical disease.^2^ The presence of multiple islet cell Aab (2 or more) is predictive of almost lifetime certainty of diagnosis and insulin requirement, making Aab an attractive target for screening programmes.^3^

The UK's National Screening Committee state a screening test should be “*simple, safe, precise and validated”* and *“from sample collection to delivery of results, should be acceptable to the target population*”.^4^ This requires a sampling option that is acceptable for use in children and is cost‐effective and a test that is suitably sensitive and specific. Type 1 diabetes Aab testing currently requires a venous blood draw which can be distressing for children, requires trained phlebotomists and carries a high failure rate (18%).^5^ Dried blood spot (DBS) capillary sampling offers an attractive alternative, as this technique is relatively simple, inexpensive, samples are stable once dry for weeks‐months, can easily be collected at home or in community settings and posted back to testing laboratories.^6^ International policymakers have called for continued development and standardisation of screening assays^7^ as consensus is lacking on the optimal screening test for T1D with advantages and disadvantages for the various platforms currently deployed for testing (Table 1).

**TABLE 1:** A comparison of platforms that measure islet cell autoantibodies.

	Advantages	Disadvantages
Radiobinding assays (RBA)	Gold standard assay for islet Aab detection High sensitivity	Requires radioactive substances making the assay unsuitable for many clinical laboratories and difficult to achieve high throughput for population screening
Electrochemiluminescence (ECL)	Can be adapted for high throughput and provides good analytical performance	Current assays require specialist reagents and platforms which are for research use only
Antibody Detection by Agglutination by PCR (ADAP)	Provides high sensitivity (particularly for IAA and GADA).^8^ Small sample volumes required Can be performed on a fully automated robotic liquid handling platform enabling high throughput	The investment required in the instrumentation and maintenance is high which is unsuitable for many healthcare economies
Luciferase immunoprecipitation (LIPS) assays	Can detect very low levels of autoantibody exceeding the sensitivity of RBAs or ELISAs.^8^, ^9^, ^10^, ^11^ High specificity Multiplexing capacity	Instrumentation such as luminometer is needed which is not available in all laboratories. Less standardisation across laboratories as fewer commercial and standard protocols exist, thus can affect reproducibility. Potential for non‐specific binding.
ELISAs	High specificity Low cost High‐throughput processing available	Islet Autoantibody Standardization Program (IASP) workshops have found ELISAs to be individually less sensitive than some other platforms If robotics used for high throughput, investment required for instrumentation and maintenance.
Multiplex ELISA—RSR 3‐screen	Sensitivity and specific in external quality assurance programmes Overall sensitivity improved with multiplex Aab testing Further cost reductions High‐throughput testing options available and can be automated for mass screening	Measurement of GADA, IA‐2A and ZnT8A but not IAA. If robotics used for high throughput, investment required for instrumentation and maintenance.

*Note*: Advantages and disadvantages of currently available assays for islet‐specific autoantibody detection.

Multiplex assays (defined here as detection of 1 or more islet autoantibodies simultaneously in one test) are being increasingly used in T1D screening and offer high‐throughput and low‐cost diagnostic platforms. Of these, the most widely available is the islet cell Aab Enzyme‐Linked Immunosorbent Assay (T1D) (3‐Screen) (RSR Limited, Cardiff, UK),^12^ used in the German Fr1da study and the European EDENT1FI study (https://www.edent1fi.eu/) which in total have screened >200,000 children to date.^13^ This assay has demonstrated high sensitivity and specificity through participation in external quality assurance programmes such as the Islet Autoantibody Standardisation Program (IASP)^14^ and UK National External Quality Assessment Scheme (NEQAS),^15^ which provide both internationally and nationally recognised benchmarks for autoantibody assay performance. IAA cannot be detected as part of the 3‐Screen. IAA ELISAs lack sensitivity and there is interference when a patient has commenced insulin treatment. Therefore, they have not been included in multiplex testing historically. However, the strategy in these studies is to identify individuals with at least two or more islet cell Aab, as they have a near 100% lifetime risk of T1D.^3^ This approach is based on the observation that individuals with only a single Aab have a 24% chance of reversion to Aab negativity.^16^ Reversion rates are also highest for IAA thus making IAA single Aab positivity of less significance than GADA or IA‐2A.^16^ Any positivity on the 3‐Screen triggers a confirmatory venepuncture sample to enable individual testing of all four Aab including GADA, ZnT8A, IA‐2A, and IAA for risk stratification. While DBS have been used successfully to detect Aab^17^, ^18^ they have not yet been verified as a sampling option for the 3‐screen.

The assay has been increasingly used by multiple laboratories, reflecting its growing popularity and utility in both research and routine clinical screening. Although automation requires an initial investment in robotic platforms, the result in high‐throughput capacity offers a cost‐effective approach to population‐level screening with an overall reduction in staff time and operator errors and can be automated for mass screening, reducing the need for expensive platforms.

The UK's EarLy Surveillance for Autoimmune diabetes (ELSA) study is exploring the acceptability and feasibility of screening children for T1D.^19^ The study protocol uses DBS sampling and the 3‐screen assay for Aab screening.^12^ We report the validation of DBS sampling, compared with serum, for the 3‐screen assay using well characterised samples of adults and children with known T1D and healthy controls. We also report the first acceptability data from semi‐structured interviews for DBS as a sampling technique for T1D screening.

## 2 METHODS

### 2.1 Recruitment and sample characteristics

Participants with known T1D, according to Paediatric and Adolescent Diabetes (ISPAD) criteria,^20^ were recruited from adult (University Hospitals Birmingham NHS Foundation Trust (UHB)) and paediatric UK diabetes clinics (Birmingham Women's and Children's NHS Foundation Trust and UHB) national research ethical approval (IRAS: 302191). Healthy control (HC) samples were obtained from laboratory donors aged ≥18 years (ethical approval from University of Birmingham; ERN_17_213). An additional *n* = 22 HC sera were purchased from Cambridge Biosciences (age range 18–60 years). The 3‐screen manufacturer claims a ≤10% positive rate in coeliac disease, ≤5% positive rate in rheumatoid arthritis, and ≤5.6% rate in Graves' disease. Surplus (*n* = 40) clinical serum samples were collected from 10 individuals with each of these conditions to assess for Aab interference. To assess precision, we acquired samples from four distributions for the Diabetic Markers scheme, from the UK National External Quality Assessment Service (NEQAS).^15^ As Aab are known to wane from time of diagnosis, a sub‐analysis of performance was undertaken according to age.

### 2.2 Sampling methodology

Finger‐prick lancets (Becton Dickinson Blue blade (1.5 mm x 2.0 mm) or Unistik© 3 Comfort 28 Gauge lancet 1.8 mm depth penetration) were used to collect capillary blood samples which were spotted onto DBS cards manufactured by Ahlstrom‐Munksjö (Helsinki, Finland). These are clinical grade with five individual pre‐perforated spots (each 12 mm in diameter containing 50 μL of dried blood at full saturation). DBS cards were stored in sealed plastic bags with desiccant at room temperature until processing. DBS samples were eluted overnight in 250 μL of 0.05% Phosphate‐Buffered Saline (PBS) Tween‐20 (Sigma), per perforated spot, representing a 1:10 dilution. The following morning, tubes were centrifuged at 10600 × *g* for 10 min and the eluate harvested for immediate testing in the 3‐screen assay. Serum was collected by venepuncture in clot activator and gel vacutainer bottles (Greiner Bio‐One, USA), processed and stored at −20°C and thawed prior to analysis.

### 2.3 Autoantibody testing

Serum and DBS eluates were tested using the 3‐screen assay as per the manufacturer's protocol^12^, ^21^ and automated using a Dynex DSX® analyser (Dynex Technologies, USA). The cut‐off for positivity is ≥20 units (U) /mL. Sensitivity and specificity for serum and DBS were calculated from the results generated from the T1D cohort and Healthy controls. These were then compared with the manufacturer claims (clinical sensitivity 86%, specificity 97%) for serum. Positive predictive value (PPV) was calculated as the total number of clinically true positive samples divided by the total number of individuals who tested positive. Negative predictive value (NPV) was calculated by dividing the number of true negatives by the total number of individuals who tested negative. For this verification, intra‐assay precision was established by running 5 serum samples five times on the same run and inter‐assay using a positive control from the 3‐Screen kit across 109 different ELISA runs, and the coefficient of variation (CV) calculated for both. The manufacturer claims an intra‐assay CV of 5.4% and inter‐assay CV of 4.4%. DBS performance was then compared with serum. Stability of dried spot cards was assessed through repeat testing and comparison of results.

It can be challenging to determine what is a true positive or negative result for three reasons: T1D patients may be positive to any of the four Aab being measured, in any combination, but may also be negative because Aab levels have waned with time since diagnosis,^22^ and 15% of individuals (idiopathic T1D) never have one of these four Aab detectable.^23^, ^24^, ^25^, ^26^, ^27^ The impact of age < or >18 years was explored and to help define Aab positivity we also undertook single Aab assay testing for IA‐2A, GADA,^28^ ZnT8A^29^ (Euroimmun for all three). Insulin (IAA) (Launch Orgentec IgG ELISA)^30^ was used to ensure the negative controls were negative for all 4 commonly report Aab but as all the T1D patients were on insulin, which can interfere with the assay, we do not report results from this cohort.

### 2.4 Statistics

Comparison of performance characteristics for the 3‐Screen using serum and DBS samples was calculated using Analyse‐it in Microsoft Excel. These were then compared with the manufacturer's serum claim and include analysis of sensitivity, specificity, negative and positive predictive value (NPV, PPV). Intra‐ and inter‐assay precision was calculated as the coefficient of variation (ratio of standard deviation to mean and expressed as percentage). Quantitative agreement between matched serum and DBS ELISA results was assessed by determining Spearman's rank correlation coefficient using GraphPad Prism version 8.0 software. This was the sample matrix comparison describing agreement between serum and DBS results using the Bland–Altman method. A *p* value <0.05 was considered statistically significant. Graphs were prepared in GraphPad Prism version 8.0.

### 2.5 Acceptability of DBS sampling

To assess the acceptability of DBS sampling, we recruited parents of children aged 3–13 years and professional stakeholders as part of the ELSA‐1 study^31^ which is exploring attitudes towards T1D screening. Purposive sampling aimed to recruit 20–30 parents and 10–20 stakeholders across diverse demographic sub‐sets (age, sex, ethnicity, with or without a first degree relative with T1D i.e. FDR or non‐FDR) to achieve thematic saturation of expressed views. The qualitative study was approved by the Greater Manchester South Health Research Authority (IRAS: 294654).

**FIGURE 1:**
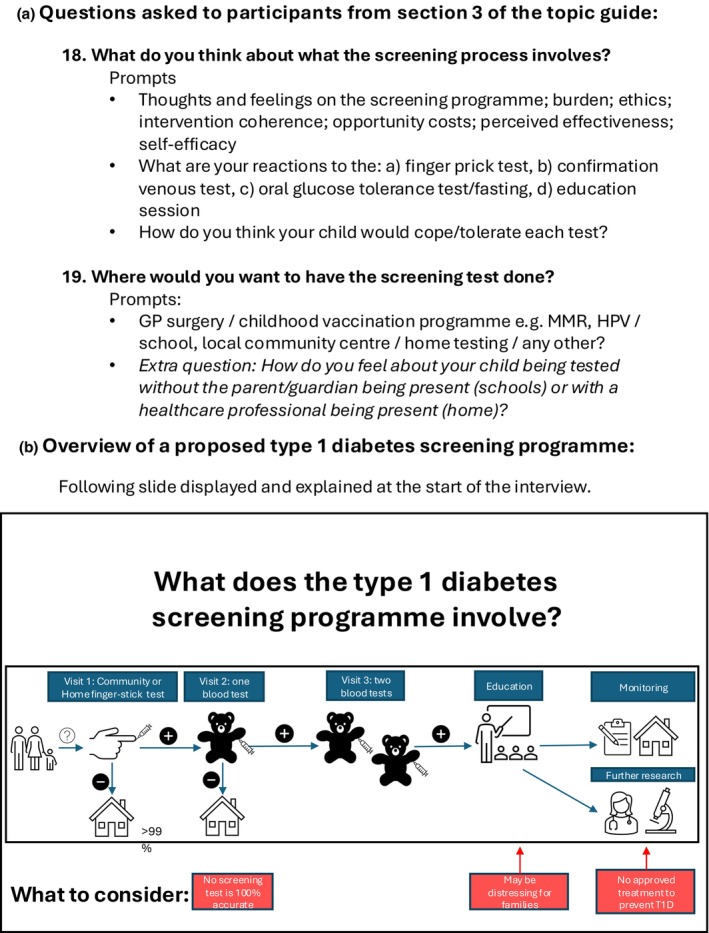
(a) Section 3 of the Topic guide from the ELSA‐1 study, including the questions pertinent to home or community DBS sampling undertaken. (b) Summary of the proposed type 1 diabetes screening programme, showing all the stages of screening. This image was provided to participants at the start of the semi‐structured interview.^31^

A 3‐min video shown at the start of the interview provided an introduction to paediatric T1D screening and an overview of the proposed screening process for context (Figure 1). Data were collected through virtual, semi‐structured interviews with parents, children, and professional stakeholders (L.M.Q.). Interviews were audio recorded and transcribed verbatim by a third party. Transcripts were analysed by two independent researchers, and codes were generated to group text. Codes were refined to synthesise themes, and inductive thematic analysis was performed to explore views towards DBS testing, preferences regarding practical aspects of testing, and any perceived barriers or facilitators to testing.

## 3 RESULTS

### 3.1 Cohort and sample description for 3‐screen DBS performance

Paired serum and DBS samples were collected from 80 adults and 19 children with T1D (age range 7–73 years) (Table S1). The median time since diagnosis for the diabetic patients was 10 years (range 1 month to 55 years). Time since diagnosis was negatively correlated with U/mL value on the 3‐Screen (Serum *r* = −0.395 [−0.55 to −0.21], *p* < 0.0001; DBS *r* = −0.244 [−0.43 to −0.04], *p* = 0.0156) (Figure S1). For the adults, the average age was 37.5 years (SD 14.4), time since T1D diagnosis was 17.3 years (SD 14.2), 76.9% were Caucasian and 48.7% female. The average age of the children was 13.8 years (SD 3.0), time since T1D diagnosis was 4.2 years (SD 3.0), 80% were Caucasian and 40% female. Two additional paediatric DBS samples were also available but without paired serum (total DBS samples *n* = 101). All DBS samples returned sufficient sample for analysis.

Healthy control (HC) samples were obtained from 10 paired serum and DBS adult laboratory donors with 17 additional DBS samples (total DBS HC = 27) (ethical approval from University of Birmingham; ERN_17_213). Additional HC serum was acquired from 22 UK healthy blood donors (Cambridge Biosciences) and 10 local laboratory donors (total serum HC *n* = 32). The average age for the combined HC cohort was 38.6 years (SD 11.8); 53.5% were Caucasian and 41.9% female (Table S1).

### 3.2 3‐screen assay performance for serum

Before determining the performance of DBS as a sampling technique, we undertook a verification of serum on the 3‐screen to enable comparison. The assay was 86% sensitive for detecting T1D in known patients (*n* = 85/99) with 97% (*n* = 31/32) specificity. From established clinical information, the PPV was calculated at 99% and NPV 69%. Serum testing met or surpassed the manufacturer's performance with the exception of a lower NPV (Table 2). However, this was not unexpected as many of the T1D samples were from individuals who have been diagnosed for many years, and it is well described that Aab titres decline over time.^22^

**TABLE 2:** Comparison of performance characteristics for the 3‐Screen using serum and dried blood spot (DBS) sampling.

Performance characteristic	Manufacturers' claim (serum only)	Serum	DBS
Clinical sensitivity	86.0%	86.0% (*n* = 85/99)	89.1% (*n* = 90/101)
Clinical specificity	97.0%	97.0% (*n* = 31/32)	100.0% (*n* = 27/27)
PPV	79.0%	99.0%	100.0%
NPV	98.0%	69.0%	71.0%
Intra‐assay CV	5.4%	4.1%	7.3%
Inter‐assay CV	4.4%	1.7%	8.1%

*Note*: Serum and DBS performance results are described and compared with the manufacturer's claims.

Abbreviations: CV, coefficient of variation; NPV, negative predictive value; PPV, positive predictive value.

Cross‐reactivity studies found no unexpected reactivity for tissue transglutaminase (TTG), thyroid peroxidase (TPO) or anti‐thyroid stimulating hormone (TSH) receptor Aab (*n* = 10 each). We detected one unexpected rheumatoid factor (RhF) positive sample (>2000 U/mL) out of 40 samples (2.5%), which met the manufacturer's claim of 5.6%. NEQAS external quality assurance found our testing to be in consensus for all four returns (*N* = 4 positive samples were distributed within a targeted range from 32.7 to 1203 U/mL).

### 3.3 Comparison of the 3‐screen serum results with single autoantibody testing

Of the 99 samples from the T1D cohort, 85 were positive on the 3‐screen ELISA. There were 67 samples that were positive on the 3‐screen and at least positive for one of the 3 corresponding Aab. Of these, 27 samples were positive for 1 Aab, 30 for 2 Aab and 10 for all 3 Aab (Figure S2 for detail). There were 18 serum samples that were positive on the 3‐screen (all positive on paired DBS) that were negative for all 3 Aab. Of the *n* = 14 T1D samples that were negative, all were negative for ZnT8A and IA‐2A; 9 samples were available for GADA and all were negative.

### 3.4 3‐screen assay performance for DBS and comparison with serum

**FIGURE 2:**
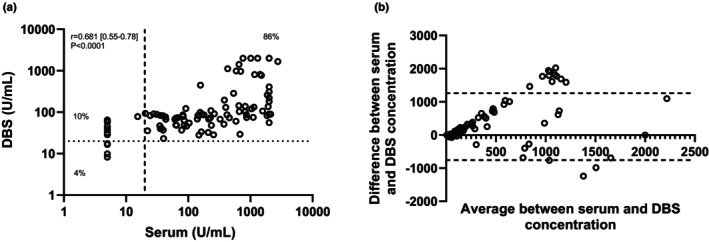
Comparison of Islet cell 3‐Screen results between serum and dried blood spots in patients with known T1D. (a) Comparison of the actual 3‐Screen values for serum and dried blood spot (DBS) analysed by Spearman correlation between *n* = 99 paired serum and DBS 3‐Screen results (*r* = 0.719 (0.60–0.80), *p* < 0.0001). Dashed lines indicate assay cut‐off value between positive and negative (≥20 U/mL). The percentage of results that fall in each quadrant is stated. The dynamic range of the 3‐screen ELISA is 5–2000 U/mL. (b) Bland–Altman plot describing agreement between concentrations (U/mL) using the two different sample types tested using the 3‐Screen, from *n* = 99 participants with type 1 diabetes (279.3 [Standard deviation ±632.1, 95% Limits of agreement −959.6‐1518]).

Clinical sensitivity (89.1% vs. 86%) and specificity (100% vs. 97%) were higher for DBS than for serum when comparing paired samples (Figure 2). Results were similar for PPV (100% vs. 99%) and NPV (69% vs. 71%). The intra‐ and inter‐assay CV was higher for DBS than for serum (7.3% vs. 4.1% and 8.1% vs. 1.7% respectively) (Table 2).

There was 94% (*n* = 93/99) concordance between paired serum and DBS samples from the T1D cohort based on the assay cut‐off of >20 U/mL. 84 paired samples were both positive, 9 both negative, 1 was positive in serum but negative on DBS, and 5 were positive on DBS where they were negative on serum. There was 100% concordance for the HC cohort (*n* = 10 serum and DBS samples were negative). Together, these gave an overall concordance of 97% between serum and DBS using the 3‐screen.

There was a strong significant correlation between DBS and serum sample values from the 3‐Screen assay (*n* = 99, *r* = 0.719 [95% CI 0.60–0.80], *p* < 0.0001) (Figure 2a) with good agreement between the matrices (Bland–Altman 279.3 [Standard Deviation ±632.1, 95% Limits of Agreement: 959.6–1518]) (Figure 2b).

Stability of DBS was assessed in 15 samples, across the dynamic range of the assay, where new spots were punched from the original card, which had been stored at room temperature with desiccant, and processed between 28 and 415 days after the initial sampling. 93.3% (*N* = 14/15) of results were concordant (sample result range: 5.9–502.4 U/mL). The discordant sample returned a low positive result (53.9 U/mL) initially and resulted in a negative result, below the 20 U/mL cut‐off, when repeated 36 days later.

### 3.5 Impact of age on performance of the 3‐screen assay

Clinical sensitivity for adults was 85.0% (*n* = 68/80) for serum and 86.4% (*n* = 70/81) for DBS samples. The mean Aab titre was 408.65 U/mL (range 15.38–2000 U/mL) for serum and 149.04 U/mL (range 8.2–1671.06 U/mL) for DBS samples. Clinical sensitivity for children was 89.5% (*n* = 17/19) for serum and 100.0% (*n* = 20/20) for DBS samples. The mean Aab titre was 695.2 U/mL (range 63.1–1931.0 U/mL) for serum and 238.4 U/mL (range: 54.8–1419.3 U/mL) for DBS (Figure S3).

### 3.6 DBS acceptability

Thirty‐eight parents and 13 children from 33 families were interviewed (5/33 interviews had 2 parents and 10/33 interviews had children present) (Table 3). Experience of T1D was documented as this may influence acceptability. Nineteen health care professionals (HCP) and six community stakeholders, including a practice manager, headteacher, school nurse, expert patient with diabetes, screening study administrator, and a diabetes clinical service coordinator, were also interviewed (Table 3).

**TABLE 3:** Demographic characteristics for acceptability study.

	Parents	Children	Professional stakeholders
Number interviewed	38 (33 families)	13	25
Age (median, range in years)	38 (25–49)	8 (5–12)	53 (39–67)
Self‐identified sex (%, female)	74%	38%	56%
Self‐identified ethnicity (%, Non‐White ethnicity)	47%	9%	20%
Socioeconomic class (National Statistics Socioeconomic Classification, most deprived, %)	24%		Not collected
First degree relative (FDR) with type 1 diabetes	30%	27%	Not collected
Occupation (% working in healthcare)	42% working in healthcare	Not applicable	76% working in healthcare 19 healthcare professionals and 6 community stakeholders. There were 6 national policymakers
Interview duration (median minutes, range)	54 (37–87)		58 (38–67)

*Note*: Demographic characteristics of the parents, children and professional stakeholders who participated in the ELSA‐1 study semi‐structured qualitative interviews.

We identified five themes from the transcripts relating to DBS testing (Table 4). Supporting quotes from parents, children and stakeholders and the interviewee characteristics are provided in the following text.

**TABLE 4:** Summary of themes and sub‐themes about the practical aspects of DBS testing from the qualitative interviews with parents and professional stakeholders.

	Theme	Sub‐themes
1	Convenience and ease of DBS testing	DBS testing considered minimally invasive, quick and easy Home‐testing most convenient
2	Finger‐prick testing experience increased parent's confidence in home‐testing	Discomfort from the finger‐prick Parental experience with finger‐prick testing increased confidence in successful completion of the DBS at home
3	Anxiety associated with screening at home (by the parent)	Fear of incorrect test completion or obtaining inadequate blood sample Sanitary issues and risk of safeguarding concerns Fear of causing pain or distress to the child Increase confidence by providing detailed instructions and videos All these concerns were resolved if DBS testing offered by a HCP in a community setting
4	Community DBS testing to improve accessibility and uptake	School clinics offered a comfortable, non‐clinical environment for DBS testing Pre‐school children would benefit from an experienced HCP performing the screening test
5	Issues with DBS testing in community settings	At school, parent not present to comfort child and no legal parent/guardian oversight Potentially distressing to combine DBS with childhood immunisation Need to explore feasibility of combining screening with childhood immunisation Home‐testing considered a convenient alternative

#### 3.6.1 Theme 1: Convenience and ease of DBS testing

Both parents and stakeholders thought DBS testing offered a quick, easy and minimally invasive screening test.

it's quite good how it starts with just a finger‐prick, because that's really, it's a cheap option, and it's not very invasive is it let's face it? [A027, Mother, non‐FDR].

Parents and stakeholders felt home‐testing was most convenient and would remove geographical, resource and social barriers to screening, such as time off from work or school.

the main benefit of the home test is that you can do it whenever you want, you don't have to take time off to have an appointment. [A010, Parent with T1D].

you would have a postal service, something comes in the post, you prick your finger, get a drop of blood on it, send it back in the post, has nothing to do with local services. [B024, Community diabetes nurse].

#### 3.6.2 Theme 2: Finger‐prick testing experience increased parents' confidence in home‐testing

Many parents interviewed were familiar with finger‐prick testing and knew it could be uncomfortable or painful, and this was recognised more so for younger children:

12‐year old boy: “just like a tiny little needle that they just poke your finger with,*”* [A024, non‐FDR child]

5‐year old girl: “It would hurt a lot.” “I think I would cry”, [A020, non‐FDR child].

However, parents with experience of finger‐prick testing recognised the discomfort would be short‐lived and were confident they could successfully perform DBS testing at home.

with a child already with diabetes that's finger pricking all the time it's absolutely fine because you do it on a regular basis, [A002, Mother of a child with T1D].

#### 3.6.3 Theme 3: Anxiety associated with screening at home (by the parent)

Parents who were unfamiliar with finger‐prick testing worried about completing the home test correctly. They feared obtaining inadequate blood samples and the need for repeat testing.

it would be a little bit more nerve wracking and I suppose unfamiliar knowing what to do and how to do it, and that might rub off on the child, [A022, non‐FDR Mother].

Parents sought clear instructions and preferred a video step‐by‐step guide, to facilitate successful completion of the home test:

if you can see a child having it done and it's not a problem then it makes you think oh yeah that's not too bad. [A002, Mother of a child with T1D].

Additional concerns with home‐testing from non‐FDR parents (*n* = 4) included sanitary issues e.g. risk of blood spillage or potential safeguarding concerns raised against the parent.

he worries if anything happens wrong like with the needle, with how the system works, maybe children going to school and speak oh my dad did this, and it will be taken in a different way of understanding. [A034, non‐FDR Father with an Arabic translator].

All parents worried about causing distress to their child in the home setting, which was eliminated if a HCP performed the screening test.

“it's really difficult for a parent to do it to their own children, especially when the child is older, you need to literally physically restrain them.” “it's better for us not to be doing it, and even better when we don't have to see it done.” [A018, non‐FDR Mother].

#### 3.6.4 Theme 4: Community DBS testing to improve accessibility and uptake for screening

Both parents and stakeholders thought screening in community settings, including schools or alongside childhood immunisation, would improve awareness, accessibility, and uptake compared to home‐testing.

other places where they're more likely to be receptive to it, so community centres, or what are the settings where they may attempt to go regularly, nurseries and schools obviously. [B002, General Practitioner (GP)].

A unique benefit of school clinics was the opportunity to test children amongst their peers in a non‐clinical environment.

I feel more confident doing it when everybody has done it, and I look up to them. [A022, 10‐year‐old boy]

The community stakeholders interviewed (*n* = 6) were supportive of school screening clinics because of positive experiences with human papillomavirus school vaccination clinics.

Allowing somebody to come in and do research, talk to families, and getting families involved in that, we [school] would support that because that's an easy thing for us to make happen, [B022, Headteacher].

Testing alongside childhood immunisation was considered most beneficial by parents of pre‐school children.

if that was alongside her vaccine that's just one less thing I need to worry about, [A021, non‐FDR Mother].

Parents preferred a HCP to test young children to address the unique challenges affecting this age group, including distress and need for ‘restraint’.

had his jabs at the GP with the practice nurse, he was absolutely fine. She literally gave him a pencil, told him he would get superpowers and we walked out, [A027, non‐FDR Mother].

#### 3.6.5 Theme 5: Issues with DBS testing in community settings

Parents raised concerns about testing young children in schools because they could not be present to physically comfort their child.

secondary school he would be fine, but in primary school they might cry, and then you don't want them crying at school. There are numerous levels of it being bad, like the teachers can't comfort them, [A009, non‐FDR Mother].

For pre‐school children, parents worried about additional distress from combining the screening test with the childhood immunisations, and GPs worried this may reduce the efficiency of a busy immunisation service.

I would rather a different appointment, because the worst thing about the vaccines is when they get to the second vaccine in the same appointment, and they are screaming already. [A009, non‐FDR Mother].

there's already a backlog of amount of work that you want to put through [general practices]. [B008, GP].

There was concern about the time needed to inform parents about screening. Information leaflets and accessible resources for more information would be essential to facilitate informed consent.

So as long as there's a recourse to someone or somewhere to ask perhaps questions that aren't answered on the initial information. [B002, GP].

## 4 DISCUSSION

We demonstrate that the 3‐screen assay is suitable for use with DBS sampling and is comparable to serum for islet‐specific Aab detection. Qualitative interviews with parents, children and stakeholders demonstrate that paediatric DBS testing was felt to be an acceptable sampling technique for T1D screening. Whilst home‐testing was most convenient, HCP community testing in schools or alongside childhood immunisation was an acceptable alternative.

### 4.1 DBS performance as a screening test

When considering the suitability or performance of a test, it is important to consider the pre‐analytic processes including sampling and transport to the laboratory as these can impact test performance. The cost and difficulty of bleeding children has already been highlighted as a major obstacle for screening children and thus, capillary sampling is an attractive alternative. The 3‐screen assay has previously been verified for use on capillary blood^21^ for the Fr1da study^13^ but this was using capillary tubes with different methodology for processing compared with DBS; therefore, it was necessary to undertake a separate verification.

DBS sampling offers numerous advantages. Firstly, it requires lower blood volumes (50 μL), compared with capillary tubes (200‐250 μL) or venous sampling (1‐2000 μL), which minimises adverse events and insufficient sample collection.^32^ In our study, DBS sampling success was 100%, whereas venepuncture was unsuccessful in two cases. A further significant advantage is the stability of the sample once it has dried, enabling community sampling at flexible times in various environments and transported using routine postal services without significant sample degradation. Finally, DBS sampling is less susceptible to haemolysis, which is a leading cause of sample rejection from capillary or serum samples in children.^33^

An international consensus workshop in 2015 (Islet Autoantibody Standardization Program, IASP), published the relative performance of different platforms and assays, and the 3‐screen, using serum, achieved 94% sensitivity and 95.6% specificity^21^ on a specific sample set. In our study, we confirmed that DBS had similar sensitivity and specificity to serum, which is consistent with other studies that have compared these matrices.^17^, ^34^ We found excellent qualitative concordance (agreement between positive or negative result) between DBS and serum. However, when compared quantitatively, values for DBS were consistently lower than those for serum. Whilst we did not find a significant difference in lower‐end assay sensitivity, there may be further optimisation gains in processing the DBS cards, as we did find more variation in DBS precision compared to serum (percentage CVs were higher). Whilst this study demonstrates the suitability of DBS for use in a screening study, further assessment is required to see whether DBS can be used for individual Aab assessment for risk stratification. A confirmatory venepuncture can also offer the advantage of early clinical contact and assessment of other biochemical parameters. Incomplete DBS spot coverage and variable blood saturation can affect precision, but these can potentially be overcome with sensor‐driven automated DBS punching machines where smaller punches are taken from optimally saturated areas.

Finally, a significant advantage of the 3‐screen is the ability to multiplex three of four T1D Aab in a high‐throughput, low‐cost system without need for expensive kits and machinery. It is easily performed by trained laboratory staff and is suitable for routine NHS use and rapid adoption for mass screening.

### 4.2 Acceptability of DBS sampling

Both parents and stakeholders thought DBS sampling offered a minimally invasive screening test, which could be most conveniently performed at home. Known advantages of DBS home‐testing include reduced geographical and social barriers for parents, i.e. less time off work or school and less distress for children from sampling in a familiar, non‐clinical setting.^35^ Although parents recognise short‐lived discomfort with finger‐prick sampling, home‐testing was still preferred over serum collection, and this is in agreement with other studies.^18^, ^36^, ^37^ In Trialnet, despite 43% finding that capillary sampling was more painful than venepuncture, 80–90% of parents preferred home‐testing.^37^

Acceptability studies in other settings show the main issues for parents with DBS home‐testing include fear of hurting the child and lack of confidence in sampling.^35^ Additional concerns raised by our parent cohort included lack of experience with finger‐prick testing, difficulty restraining a young child, incorrect test completion, obtaining insufficient sample, sanitary issues and safeguarding concerns. However, community testing appeared acceptable to parents who may otherwise decline home‐testing. Parents preferred testing in schools for older children who could give informed assent and were less likely to become distressed without their parent. Conversely, parents with younger children preferred screening by an experienced HCP alongside immunisations.

Advantages of community HCP testing include increased successful completion and return rates.^38^ For example, the UK Newborn Screening (NBS) program^38^ utilises a heel prick test in the first days of life which stores capillary blood within a DBS and achieves >99% uptake^38^ and ≤2% avoidable repeats.^39^ Similarly, Fr1da demonstrated a 99.5% sufficient sample return rate with capillary testing performed by a HCP and 100% of DBS cards collected in our validation study returned sufficient sample, surpassing success rates for paediatric venepuncture (82%).^13^ In comparison, only 57% of parent‐collected capillary tube collections were returned with sufficient sample in the Swedish autoimmune Triad study,^40^ whilst 93% of self‐collected DBS samples were returned with sufficient sample in the Australian T1D national screening pilot.^18^ Hence, although home testing is susceptible to unreturned samples and insufficient sample collection, this is minimised when community HCP testing is offered. Although this is more expensive and resource intensive compared to home testing, this may prove cost effective if the sampling failure rate is low. DBS failure rates in different settings will be examined as part of the larger ELSA study.

Overall, we provide the first acceptability data for DBS sampling from the general population and T1D family members. Limitations of the qualitative study include evaluation of hypothetical views towards DBS as a screening test and a third of included families had lived experience of T1D, which may have influenced acceptability. However, acceptability assessments from diverse screened cohorts are ongoing for the ELSA study.

In conclusion, utilising DBS within a multiplex Aab assay offers an accessible, acceptable, minimally invasive screening solution, with reduced blood collection volume and ease of sampling at home or in community settings. This offers potential cost savings and improved convenience for families, rendering DBS sampling well suited to any future mass screening programme for T1D.

## AUTHOR CONTRIBUTIONS

S.E.F., L.M.Q., M.H., S.Y., C.B., H.F.K., T.P., I.L., F.B., S.M.G., P.N., A.G.R. were involved in the conception, design, and conduct of the study and the analysis and interpretation of the results. L.M.Q., S.E.F. and A.G.R. wrote the first draft of the manuscript, and all authors edited, reviewed, and approved the final version of the manuscript. A.G.R. is the guarantor of the validation study and, as such, had full access to all the data in the study and takes responsibility for the integrity of the data and the accuracy of the data analysis. L.M.Q. is the guarantor of the qualitative study and, as such, had full access to all the data in the study and takes responsibility for the integrity of the data and the accuracy of the data analysis. The datasets generated during this study are available from the corresponding author upon reasonable request.

## FUNDING INFORMATION

The ELSA study is funded by Diabetes UK and Breakthrough T1D (formerly JDRF) (grant code: 20_0006315). The ELSA‐1 study was supported by a grant from the National Institute for Health and Care Research (NIHR202816). The views expressed are those of the authors(s) and not necessarily those of the NHS, the NIHR, or the Department of Health. The funders had no role in the study design; no role in data collection, analysis, and interpretation of data; no role in the writing of the protocol paper; and no role in the decision to submit the paper for publication.

## CONFLICT OF INTEREST STATEMENT

The authors declare that they have no known conflicts of interest, competing financial interests, or personal relationships that could have appeared to influence the work reported in this paper.

## Supporting information


**Figure S1.** Autoantibody levels wane over time since diagnosis with Type 1 Diabetes (T1D).

**Figure S2.** Comparison of serum 3‐screen with individual autoantibody ELISAs.

**Figure S3.** Comparison between serum and dried blood spot (DBS) in children and adults. (a) Spearman’s correlation of DBS and serum concentrations from the 3‐screen ELISA in paired samples from *n* = 19 children with T1D (*r* = 0.760 (0.46–0.91), *p* = 0.0002). (b) Spearman’s correlation of DBS and serum concentrations in paired samples from the 3‐screen ELISA in *n* = 80 adults with T1D (*r* = 0.705 (0.57–0.80), *p* < 0.0001). (c, d) There were no significant differences in the values of the 3‐screen assay between children and adults in either serum (c) or DBS (d).

**Table S1.** Description of cohorts.
