# Supplementary figures and images for: Establishing the performance and acceptability of dried blood spot sampling to screen for islet‐specific autoantibodies
Source: Diabet Med. 2025 May 19;42(8):e70071. doi: 10.1111/dme.70071 (PMC12257432; doi:10.1111/dme.70071)

**Supplementary Figure 1: Autoantibody levels wane over time since diagnosis with T1D**

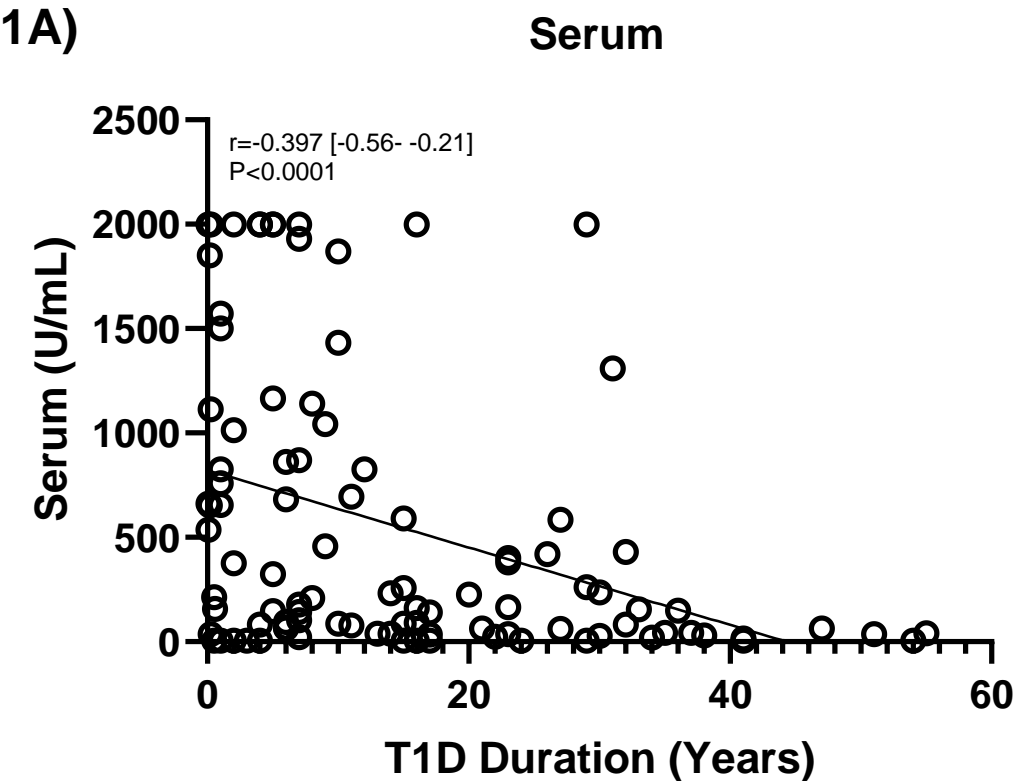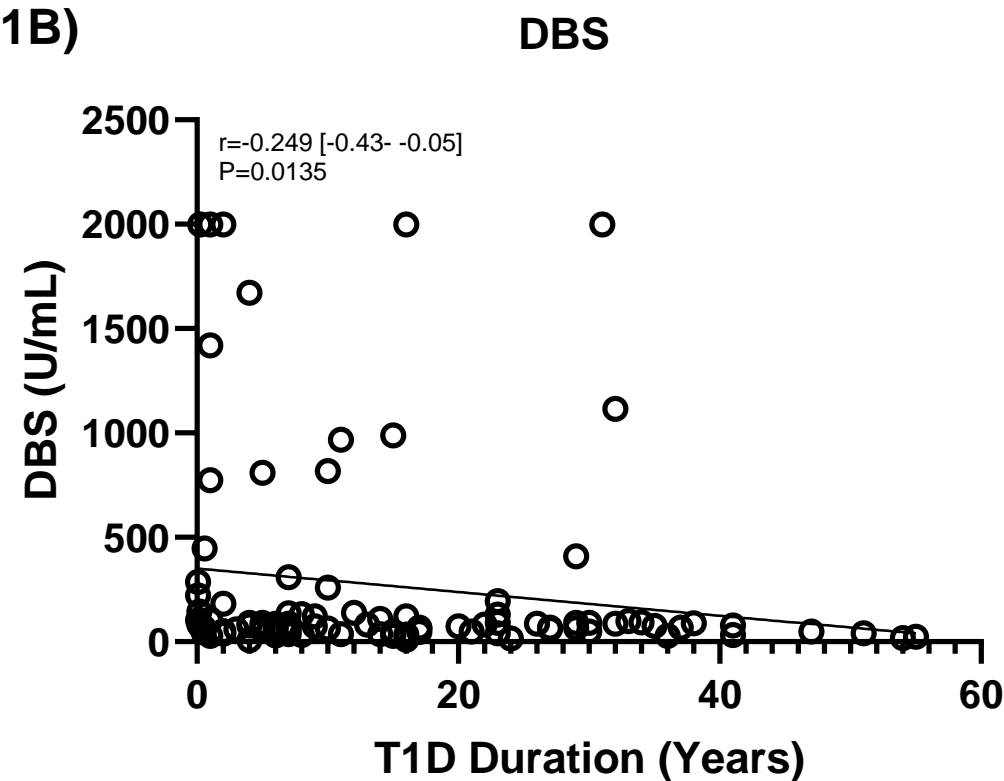

Supplement: Supplementary file 1 — Figure S1. Autoantibody levels wane over time since diagnosis with Type 1 Diabetes (T1D). [file DME-42-e70071-s003.pdf]

**Supplementary Figure 2: Comparison of serum 3-screen with individual autoantibody ELISAs**

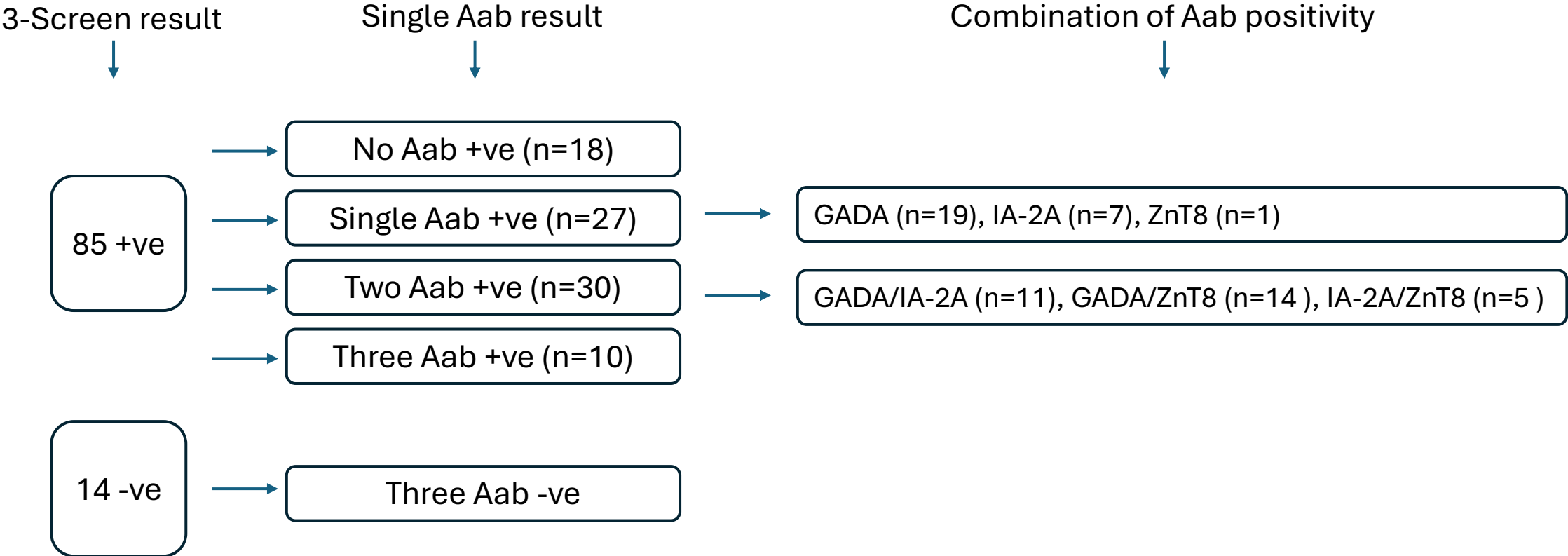

Supplement: Supplementary file 2 — Figure S2. Comparison of serum 3‐screen with individual autoantibody ELISAs. [file DME-42-e70071-s004.pdf]
