# Supplementary material for: Establishing the performance and acceptability of dried blood spot sampling to screen for islet‐specific autoantibodies
Source: Diabet Med. 2025 May 19;42(8):e70071. doi: 10.1111/dme.70071 (PMC12257432; doi:10.1111/dme.70071)

Supplementary Figure 3: Comparison between Serum and Dried Blood Spot (DBS) in Children and Adults

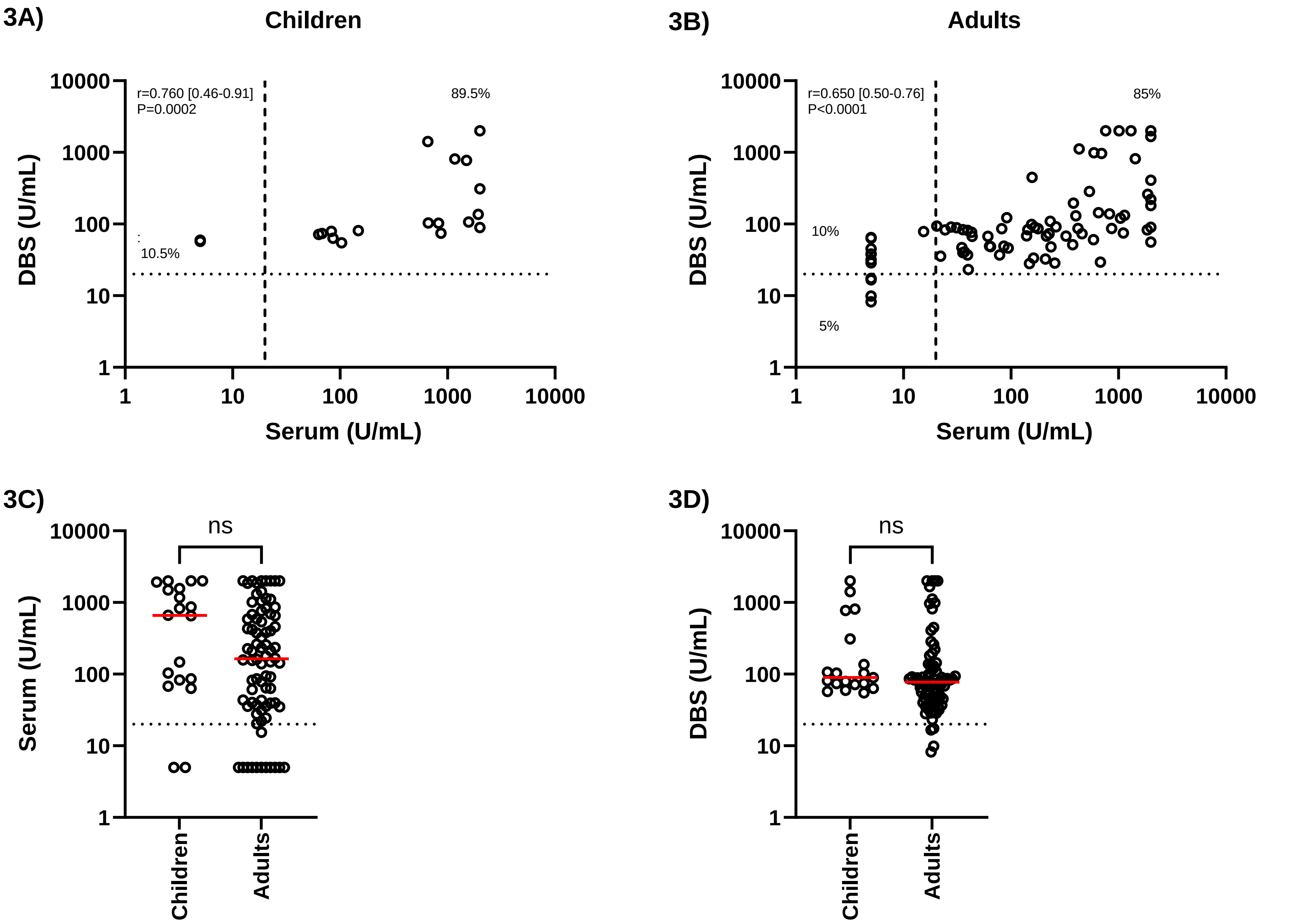

Supplement: Supplementary file 3 — Figure S3. Comparison between serum and dried blood spot (DBS) in children and adults. (a) Spearman’s correlation of DBS and serum concentrations from the 3‐screen ELISA in paired samples from n = 19 children with T1D (r = 0.760 (0.46–0.91), p = 0.0002). (b) Spearman’s correlation of DBS and serum concentrations in paired samples from the 3‐screen ELISA in n = 80 adults with T1D (r = 0.705 (0.57–0.80), p < 0.0001). (c, d) There were no significant differences in the values of the 3‐screen assay between children and adults in either serum (c) or DBS (d). [file DME-42-e70071-s002.pdf]
