# Supplementary material for: Establishing the performance and acceptability of dried blood spot sampling to screen for islet‐specific autoantibodies
Source: Diabet Med. 2025 May 19;42(8):e70071. doi: 10.1111/dme.70071 (PMC12257432; doi:10.1111/dme.70071)
Supplement: Supplementary file 4 — Table S1. Description of cohorts. [file DME-42-e70071-s001.docx]

**Supplementary Table 1: Description of Cohorts**

|  | **T1D cohort** | | **Healthy adult controls n=49** |
| --- | --- | --- | --- |
|  | **Children <18 years) n=19** | **Adults ≥ 18 years n=80** |  |
| **Time since T1D diagnosis (Years, mean (SD))** | **4.2 (3.0)** | **17.3 (14.2)** | **NA** |
| **Age (Years, mean (SD))** | **13.8 (3.0)** | **37.5 (14.4)** | **38.6 (11.8)** |
| **Sex (Female)** | **40%** | **48.7%** | **41.9%** |
| **Ethnicity (Caucasian)** | **80%** | **76.9%** | **53.5%** |

*Supplementary Table 1 legend: Gender and ethnicity is not known for 9 children and 2 adults in the T1D cohort. Duration of T1D not known for 1 child. For the healthy control cohort, age is not known for 7 and gender and ethnicity for 5 adults. Familial T1D status was not known for either the T1D nor healthy control cohorts.*
